# Transforming mental health care with a digital platform for adaptive PREMs and PROMs

**DOI:** 10.1192/j.eurpsy.2025.1569

**Published:** 2025-08-26

**Authors:** L. Boyer, P.-M. Llorca, G. Fond, Y. Brousse, S. Fernandes

**Affiliations:** 1 AMU, Marseille; 2 CHU Clermont Ferrand, Clermont Ferrand, France

## Abstract

**Introduction:**

In transforming mental health care, integrating patient perspectives is essential through Patient-Reported Experience Measures (PREMs) and Patient-Reported Outcome Measures (PROMs). PREMIUM and MyPsy&I® are two French initiatives advancing this approach by developing adaptive PREMs and PROMs and implementing a digital platform for seamless data collection, fully integrated within France’s national health information systems.

**Objectives:**

The objectives were (1) to develop eight item banks (seven PREMs and one PROM) and their corresponding Computerized Adaptive Tests (CATs) using a rigorous methodology grounded in Item Response Theory (IRT) (PREMIUM); and (2) to create a digital platform designed for multi-level use (micro, meso, and macro), enabling integration within the national health information system for enhanced patient feedback collection and analysis (MyPsy&I®).

**Methods:**

This multicenter, cross-sectional study uses a mixed-methods approach, integrating qualitative and quantitative methodologies across three main phases: (1) development of item banks and corresponding CATs based on a standardized procedure, including conceptual work, domain mapping, item selection, item bank calibration, and CAT simulations and validation; (2) development of a digital platform tailored for multi-level use and integrated within the national health information system; and (3) a qualitative study exploring the implementation and acceptability of both the CAT and the digital platform.

**Results:**

More than 3,000 patients with schizophrenia, bipolar disorder, and major depressive disorder were recruited from various inpatient and outpatient settings in France.

Unidimensionality, local independence, and monotonicity were verified for each item bank. Psychometric properties were satisfactory for both internal (RMSEA ≤ 0.08, CFI, TLI ≥ 0.95, and infit mean square statistic ranging between 0.7 and 1.0) and external validity. Each CAT demonstrated satisfactory accuracy and precision (standard error of measurement < 0.55 and root mean square error < 0.3), with an average administration of eight items.

The MyPsy&I® digital platform has shown strong acceptability and value across 12 French hospitals, facilitating efficient, real-time collection of patient feedback. This streamlined approach allows healthcare providers to integrate patient perspectives at various stages of care, enhancing both the quality and responsiveness of mental health services.

**Image:**

**Figure:**
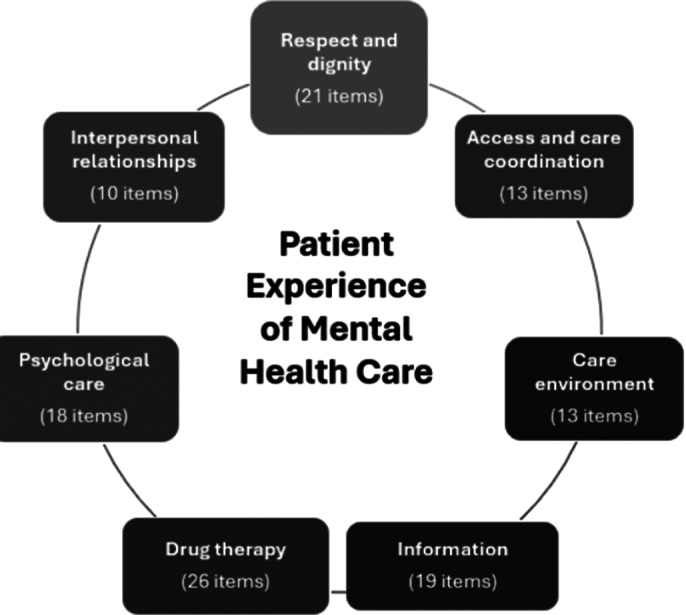

**Image 2:**

**Figure:**
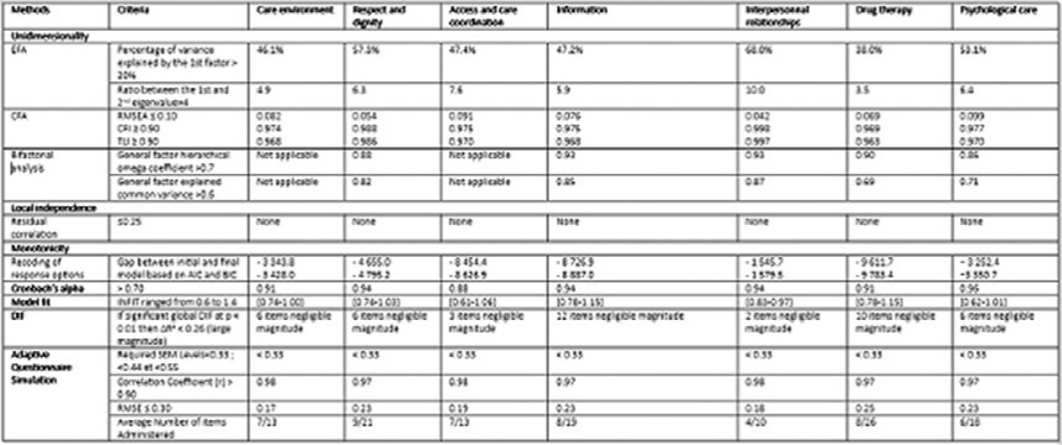

**Conclusions:**

This work provides adaptive PREMs, PROMs, and a digital platform that streamline patient feedback collection, reducing burden on patients and providers. Integrating these tools into the health information flow is essential for embedding patient perspectives in modern healthcare systems, especially in a digital and AI-enhanced environment.

**Disclosure of Interest:**

None Declared

